# Neurodevelopmental Outcomes in the Offspring of Women with Vitamin D Deficiency and Women Who Received Vitamin D Supplementation During Pregnancy

**DOI:** 10.3390/nu17060978

**Published:** 2025-03-11

**Authors:** Antonia Varthaliti, Kalliopi Rodolaki, Vasilios Lygizos, Dimitrios Efthymios Vlachos, Nikolaos Thomakos, Dimos Sioutis, George Daskalakis, Vasilios Pergialiotis

**Affiliations:** 1 Department of Obstetrics and Gynecology, “Alexandra” Hospital, National and Kapodistrian University of Athens, Vasilissis Sofias 80 Avenue, 11528 Athens, Greece; antonia.varthaliti@hotmail.com (A.V.); kelli1_@hotmail.com (K.R.); vlygizos@gmail.com (V.L.); vlachos.dg@gmail.com (D.E.V.); thomakir@hotmail.com (N.T.); gdaskalakis@yahoo.com (G.D.); 2 Department of Obstetrics and Gynecology, University General Hospital “ATTIKON”, National and Kapodistrian University of Athens, 11528 Athens, Greece; dsioutis@gmail.com

**Keywords:** vitamin D, pregnancy, neurodevelopment, cognitive function, motor development, language acquisition, maternal supplementation, fetal brain development, public health

## Abstract

Vitamin D is an essential nutrient, involved in various biological processes including calcium homeostasis, bone health, immune function, and brain development. Vitamin D from the mother crosses the placenta during pregnancy, directly impacting the neurodevelopment of the fetus. Vitamin D insufficiency is a substantial global health problem, influencing almost 47.9% of individuals, with especially high predominance rates among pregnant women. **Background/Objectives**: Preclinical studies suggest that maternal vitamin D deficiency results in significant alterations in the development of the offspring’s brain. Nonetheless, randomized clinical trials in humans have produced conflicting results on the beneficial effect of high-dose vitamin D supplementation during pregnancy on neurodevelopmental outcomes. This review aims to evaluate the association of maternal prenatal vitamin D levels and vitamin D supplementation during pregnancy with offspring neurodevelopment. **Methods**: This study thoroughly reviewed the literature and searched throughout PubMed, ScienceDirect, Cochrane Library, and Google Scholar, adhering to PRISMA guidelines. Studies assessing maternal vitamin D levels, supplementation, and offspring neurodevelopmental outcomes were included based on predefined eligibility criteria. **Results**: Among 9686 screened studies, only 20 met the inclusion criteria, representing 18,283 mother–child pairs. A small, non-significant trend suggested a positive association between higher maternal vitamin D levels and offspring cognitive, language, motor, and social-emotional development. The strongest associations were observed in cognitive performance and language acquisition, though inconsistencies emerged across studies. Cord blood vitamin D levels showed no consistent effects on neurodevelopment. Maternal vitamin D supplementation during pregnancy demonstrated no reliable benefits for offspring neurodevelopment, with results varying by study design and participant characteristics. **Conclusions**: Large-scale, multicenter randomized trials, with standardized neurodevelopmental assessments at multiple ages are needed to define the effects of vitamin D deficiency and supplementation during pregnancy on offspring neurodevelopmental outcomes. Future research should investigate the confounding factors contributing to inconsistencies, including supplementation protocols, genetic variations, and assessment methodologies. Clarifying these aspects will enhance the understanding of maternal vitamin D’s role in fetal neurodevelopment and aid in refining prenatal supplementation guidelines.

## 1. Introduction

Vitamin D is a fundamental nutrient that plays a critical role in various functions of the human body and is involved in calcium homeostasis, bone health, brain development, and immune function. During pregnancy, maternal vitamin D crosses the blood-placental barrier, and it is essential for the development of the central nervous system of the fetus [1]. Vitamin D is known to affect neuronal differentiation, axonal connectivity, dopamine ontogeny, immune modulation, and gene transcription, all of which are essential for the normal development of the brain and as a result for the more important neurodevelopmental functions in children. It acts as a neurosteroid hormone, regulating key processes that support both fetal brain development and cognitive function in later life.

Data and studies on whether deficient vitamin D levels are associated with neurodevelopmental disorders are conflicting, with some linking low vitamin D levels with poor outcomes, and others not showing significant associations [2]. It is critical that when considering the available literature we consider the type of studies since the ability to perform a randomized controlled trial is not always possible in the field of obstetrics.

Deficiency of vitamin D during pregnancy has been associated with adverse neurodevelopmental outcomes for the offspring, including impairments in cognitive, motor, language, social-emotional, and behavioral skills in children. Studies suggest that low levels of maternal vitamin D can result in increased risk of neurodevelopmental disorders such as Autism Spectrum Disorder (ASD), Attention-Deficit Hyperactivity Disorder (ADHD), schizophrenia, depression, anxiety disorders, impaired cognitive function, reduced IQ, memory, learning ability, and problem-solving skills, and delayed motor and language development. It is even believed that, beyond early brain development, vitamin D plays a neuroprotective role throughout life, reducing the risk of neurodegenerative disorders, including Multiple Sclerosis, Alzheimer’s and Parkinson’s disease. Pregnancy represents a very important period for the future development of the offspring [3]. However, there are studies that show increased rates of atopic disorders, like eczema and asthma, with higher vitamin D maternal levels, and no effect on neurodevelopmental disorders [4].

Vitamin D deficiency poses a major global public health concern, affecting nearly 47.9% of the general population [5]. This number is even higher for pregnant women, particularly in developing countries where deficiency rates range from 56% to 96%.

Animal studies suggest that vitamin D deficiency during pregnancy causes significant changes in the offspring’s brain development and contributes to behavioral impairments, affecting developmental milestones, memory, and learning abilities in childhood and adulthood [6,7]. However, randomized clinical trials in humans have found no significant benefits of supplementation of high-dose vitamin D prenatally at well-known assessment tests in children [8,9]. These findings suggest that exceeding the recommended vitamin D intake does not enhance cognitive outcomes, although they do not rule out the potential effects of vitamin D deficiency. Furthermore, observational studies over the past decade have shown inconsistent results about the relationship between maternal vitamin D status and cognitive function in children [10,11]. Direct comparison between studies comparing human and non-human subjects is not possible and it is beyond the scope of the present study; however, a few points can be extracted and noted: human brain development seems to defer significantly from a non-human brain [12], and thus vitamin D levels may or may not play an important role.

Given the potential effects of childhood development, this review aims to investigate whether there is an association between maternal prenatal vitamin D levels and vitamin D supplementation during pregnancy for shaping an offspring’s neurodevelopment, in order to implement new guidelines and strategies for optimizing maternal and offspring wellbeing. More specifically, this review will examine studies on maternal vitamin D levels and supplementation during pregnancy to assess their impact on neurodevelopment until the age of 9 years old, considering age-specific effects and differences in study outcomes.

## 2. Materials and Methods

This review is structured according to the Preferred Reporting Items for Systematic Reviews and Meta-Analyses (PRISMA) guidelines. The review is based on accumulated data previously published in international literature, so patient consent and institutional review board approval were not required. This study is registered in PROSPERO (CRD420250650549).

### 2.1. Eligibility Criteria, Information Sources, and Search Strategy

**Figure 1:**
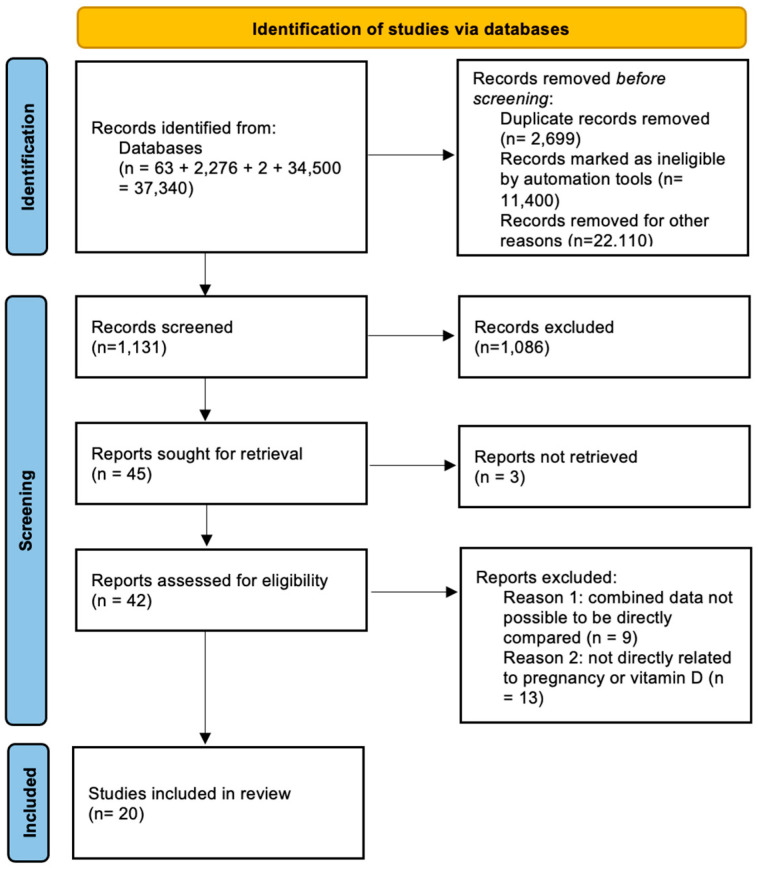
Search flow diagram based on PRISMA guidelines.

A thorough search of the literature was conducted by two reviewers A.V. and K.R. across multiple databases, including PubMed, ScienceDirect, the Cochrane Library, and Google Scholar between November and December 2024. The authors predetermined the eligibility criteria. All studies that examined the impact of maternal or cord blood vitamin D levels or vitamin D supplementation during pregnancy in children’s neurodevelopment were considered eligible for inclusion. The two independent reviewers collaboratively selected the eligible studies and reached a consensus on which studies to include. The two reviewers independently assessed the search results, and any disagreements were resolved through discussions in collaboration with a third author (V.L.) before finalizing the selected papers. Randomized and non-randomized studies, including prospective and retrospective designs, involving human patients, were considered eligible for this review. Additionally, only studies published in English were included. The present study is based on aggregated, published data and it is not an RCT. PICO criteria were included in the present revision and involved (P): pregnant women who had vitamin D deficiency, (I): vitamin D supplementation, (C): women without vitamin D deficiency who were compared to women with vitamin D deficiency as well as women that did not receive vitamin D supplementation, and (O): differences in neurodevelopmental outcomes of offspring based on standardized questionnaires, irrespective of the actual age at assessment. Case reports, experimental studies, conference abstracts, reviews, and animal studies were excluded from our study. Our search strategy included the following text terms “[pregnancy AND (vitamin D)] AND neurodevelopment” and is presented in Figure 1. The final studies included ranged from 2008 until 2024. Differences were expected among the included studies in the range of vitamin D values. All studies were selected for inclusion, considering the possibility of a potential subgroup analysis.

### 2.2. Study Selection

The selection of studies was conducted in three successive stages. Initially, duplicate articles were removed. Next, reviews and animal studies were excluded. The two authors (A.V. and V.L.) manually screened the abstracts of the remaining articles to assess their eligibility. Ultimately, studies considered potentially eligible were included after a thorough review of their full texts. Any discrepancies during this final stage were determined through agreement among all authors.

### 2.3. Data Extraction

Manuscripts describing the impact of maternal vitamin D affected by another factor or they did not have comparable data were also excluded. Finally, 20 papers were included in our review. The outcome measures were established during the initial design of this review. The primary focus of our study was to assess the influence of maternal and cord blood vitamin D levels, along with maternal vitamin D supplementation during pregnancy, on the neurodevelopment of the offspring [2] but also on social and behavioral skills, as these skills are also necessary for a person’s development.

### 2.4. Assessment of Risk of Bias

**Figure 2:**
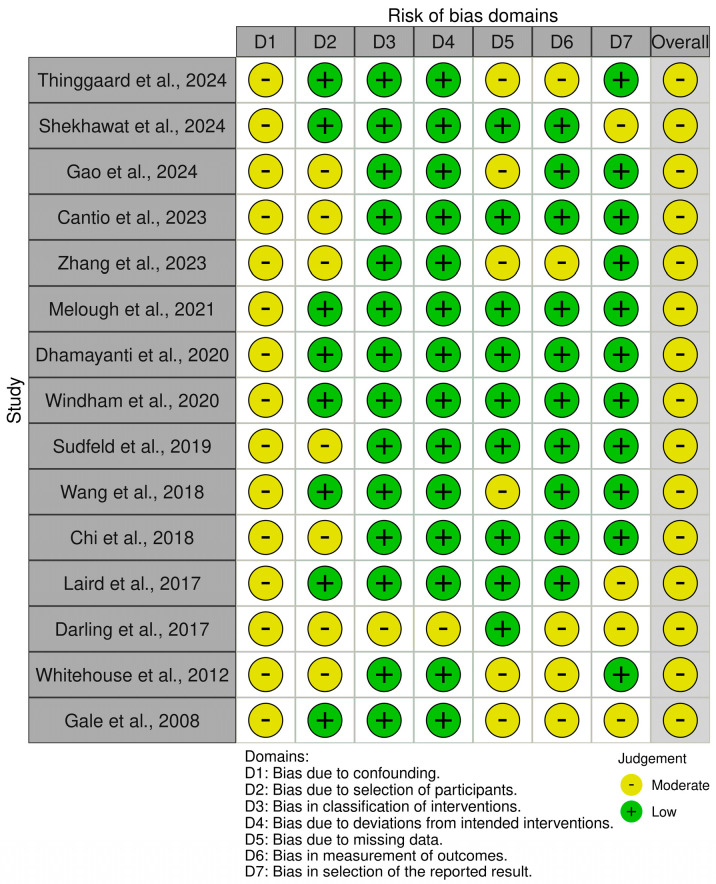
Risk of bias assessment using the Cochrane ROBINS-I tool (*n* = 15) [4,14,15,16,17,18,19,20,21,22,23,24,25,26,27].

Two authors (A.V. and K.R.) evaluated the methodological quality of the selected observational studies, using the ROBINS-I (Risk of Bias in Non-randomized Studies-of Interventions) tool [13], as shown in Figure 2, that assesses the risk of bias in non-randomized studies and evaluates the extent to which the results of a study might be affected by biases that could distort the true effect of the intervention. This tool assesses bias across seven domains: bias due to confounding, in the selection of participants into the study, in the classification of interventions, due to deviations from intended interventions, due to missing data, in the measurement of outcomes, and in the selection of reported results.

**Figure 3:**
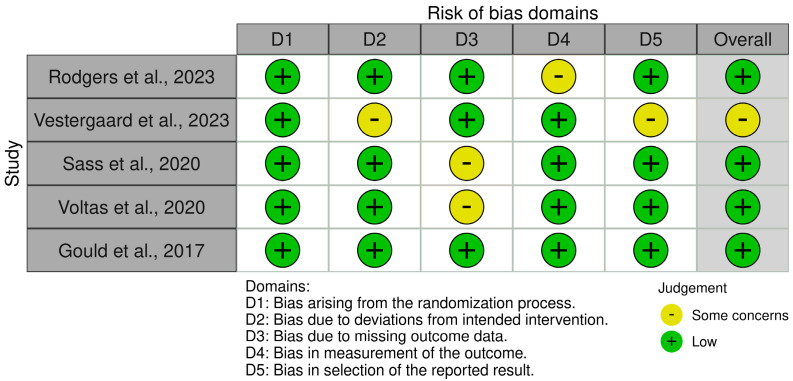
Risk of bias assessment using the Cochrane RoB-2 tool (*n* = 5) [9,29,30,31,32].

As for the assessment of randomized control trials, two authors (A.V. and K.R.) used RoB 2 tool (Risk of Bias 2) [28], as shown in Figure 3, a standardized tool to assess the risk of bias in randomized controlled trials (RCTs). It evaluates the internal validity of the study results by systematically identifying and addressing potential biases in the design, as well as conducting and reporting of RCTs. When the two reviewers differed, a final consensus was specified by a third author (V.L.).

## 3. Results

The thorough search resulted in a total of 37,340 initial records: 63 from PubMed, 2776 from ScienceDirect, 2 from the Cochrane Library, and 34,500 from Google Scholar. After eliminating 2699 duplicate entries, 11,400 reviews and 22,110 animal studies, 1131 original studies were screened based on their titles and abstracts. Out of those 1131, 1086 were excluded as they were not fulfilling the eligibility criteria (i.e., conference abstracts, case reports, and book chapters).

Finally, twenty studies were included in our review that involved 18,283 mother–child pairs [4,9,14,15,16,17,18,19,20,21,22,23,24,25,26,27,29,30,31,32], and were published between 2008 and 2024. Table 1 depicts the basic information of all the included studies. Fifteen studies (references) evaluated the relationship between antenatal maternal vitamin D levels and children’s neurodevelopment at various ages using commonly available assessment scales. In addition, three studies assessed whether vitamin D supplementation during pregnancy had an impact on children’s neurodevelopment. Furthermore, two studies examined the link between cord blood vitamin D levels at delivery and neurodevelopment in children.

**Figure 4:**
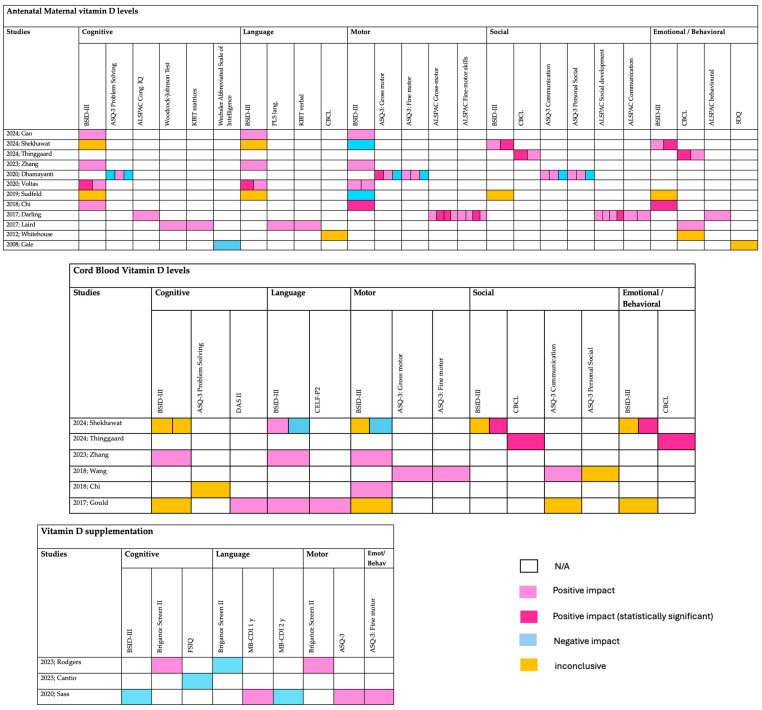
Neurodevelopmental outcomes of the included studies [4,9,14,15,16,17,18,20,22,23,24,25,26,27,29,30,32].

In this review, the most common neurodevelopmental functions are addressed: cognitive, language, motor, social, emotional, and behavioral functions, using all reporting measures, as indicated in individual studies. The results are shown in Figure 4 and show that antenatal maternal vitamin D levels had a mild positive impact on children’s neurodevelopment in some cases, which failed to reach statistical significance. Vitamin D supplementation during pregnancy correlated poorly with children’s neurodevelopmental outcomes. Cord Blood Vitamin D levels during delivery had mostly inconclusive results.

In the included studies, cognitive function is assessed through various tools, including the Bayley Scales of Infant and Toddler Development (BSID-III), Ages and Stages Questionnaires (ASQ-3) Problem Solving, Full-Scale Intelligence Quotient (FSIQ), Avon Longitudinal Study of Parents and Children Cognitive (ALSPAC Cogn.) IQ, Brigance Screen II, Woodcock-Johnson Scholastic Achievement Test, Kaufman Brief Intelligence Test (KBIT) matrices, and Differential Ability Scales-II (DAS-II). Several studies reported a positive association between higher maternal vitamin D levels and cognitive performance in offspring, particularly in IQ and problem-solving skills. One study showed statistically significant improvement, whereas others reported inconclusive or negative findings. This suggests that while maternal vitamin D plays a role in cognitive development, the results in the existed studies are inconsistent.

Language development is assessed using techniques such as BSID-III, Preschool Language Scale (PLS), KBIT Verbal, ASQ-3 Communication, MacArthur-Bates Communicative Development Inventories (MB-CDI 1 year or 2 years old), and Clinical Evaluation of Language Fundamentals Preschool-2 (CELF P-2). Most studies showed a positive impact on verbal and communication skills, but only the study from Voltas et al. reached statistical significance. Rodgers et al. [30] and Cantio et al. [18] reported positive associations between prenatal vitamin D supplementation and improved early verbal abilities. Sass et al. did not find a significant benefit. Multiple studies indicated a positive association between higher cord blood vitamin D levels and language acquisition and communication abilities, with some statistically significant findings. However, the findings remained inconclusive, suggesting that maternal vitamin D might be one of several factors affecting language acquisition.

Motor development is assessed through BSID-III, ASQ-3 (gross and fine motor), and ALSPAC Gross-Motor Skills. Most studies showed a positive relationship between maternal vitamin D sufficiency and better motor function in infants and toddlers, particularly in fine and gross motor skills. Dhamayanti et al. found statistically significant improvements in the gross motor skills at 3 months of age while did not find any improvements at 12 months of the gross and fine motor skills [20]. Chi et al. found statistically significant improvement in 6 months [23]. Sudfeld et al. reported no effect [22]. While supplementation of vitamin D appeared to support motor development, its effects were less pronounced than in other domains such as cognition and language. Rodgers et al. and Cantio et al. found improvements in motor skills, though results were not always statistically significant [18,30]. Newborn vitamin D levels showed a positive association with gross and fine motor development, but like maternal levels, the findings were inconsistent across studies. Some studies reported statistically significant benefits, while others found no clear impact.

Social development is measured using ASQ-3 Personal-Social and ALSPAC Social Development Scales. Emotional development is evaluated through CBCL (Child Behavior Checklist) and ASQ-3 Emotional Scales. Behavioral development, including potential links to ADHD and autistic traits, is assessed using CBCL, ALSPAC behavioral measures, and SDQ (Strengths and Difficulties Questionnaire). In social, emotional, and behavioral functions the results for the link of vitamin D and neurodevelopmental outcomes were also mixed.

The analysis of the risk in bias of non-randomized trials indicated a low risk of bias across key domains, including confounding, intervention classification, and deviations from intended interventions. However, moderate risk was identified in missing data, outcome measurements, and result selection. Additionally, randomized controlled trials demonstrated a low risk of bias, particularly in randomization, outcome measurement, and result selection. Nevertheless, some concerns were noted in deviations from intended interventions and missing outcome data. In addition, for missing data, we used the listwise deletion approach wherever the MCAR assumption was satisfied. Overall, the quality of the methodology of the included studies was high, as shown in Figure 2 and Figure 3, but certain limitations should be considered when interpreting the findings.

## 4. Discussion

The purpose of the present study was to systemically review and summarize the existing literature regarding the link between antenatal maternal vitamin D levels, prenatal vitamin D supplementation, as well as cord blood vitamin D levels, and offspring neurodevelopment. The results of our study indicate that the effect of maternal vitamin D levels on subsequent offspring neurodevelopment is relatively small and not statistically significant. Moreover, additional intake of vitamin D supplements during pregnancy did not seem to contribute to better neurodevelopmental outcomes. Finally, our findings from the two included studies concerning the association between cord blood vitamin D levels and neurodevelopment, did not draw definite conclusions. Based on these findings, maternal vitamin D levels and the need for vitamin D supplementation during pregnancy cannot be justified. However, since there are huge inequalities worldwide regarding the source of vitamin D during pregnancy, more thorough studies are needed in order to fully clarify these results. Of note, we probably may not be able to generalize our findings in a worldwide setting and an effect size may have an impact; however, based on the available studies, we have included subjects that can offer a meaningful insight into the current practices regarding vitamin D supplementation.

Vitamin D is an essential micronutrient with crucial implications for human health and disease. The main source of vitamin D for humans is the ultraviolet B radiation while only a small amount may be provided through dietary sources like sun-exposed mushrooms, eggs, and fatty fish. Vitamin D is hydroxylated in the liver into 25-hydroxyvitamin D (25(OH)D) which constitutes the majority of circulating vitamin D in the human body and represents the most valid indicator of vitamin D status. Vitamin D deficiency has been recognized as an important Public Health concern and it is estimated that approximately half of the population worldwide have insufficient vitamin D levels with subsequent devastating health consequences. Apart from its vital role in the regulation of calcium homeostasis and normal bone health, accumulating evidence from current research suggests that vitamin D has a profound effect on various diseases including heart-related, metabolic and autoimmune disorders, infectious diseases, and different types of cancer [1]. During pregnancy, the growing fetus is highly dependent on maternal vitamin D status. Via multiple mechanisms, vitamin D promotes the expression of NT-3, NT-4, and nerve growth factors, which are necessary during brain development.

Convincing evidence from the current literature has shown that reduced maternal vitamin D levels are an established risk factor for multiple adverse perinatal outcomes, posing a great threat to both the mother and the developing fetus. Numerous studies have shown that vitamin D deficiency significantly increases the risk for gestational diabetes mellitus, pregnancy-induced hypertension as well as spontaneous abortion and still birth. Furthermore, vitamin D insufficiency may contribute to higher rates of cesarian section delivery due to a reduction in contractile muscle strength, intrauterine growth restriction, low birth weight, and neonatal hypocalcemia [33]. Recent studies from human and animal models have highlighted the unique role of vitamin D in embryo implantation, placental formation and function as well as intrauterine regulation of inflammatory cytokine release and fetal skeletal growth, vitamin D deficiency exacerbates placental inflammation and disrupts normal placental function, and increases oxidative stress. Nevertheless, despite the great importance of vitamin D in all aspects of fetal and maternal wellbeing, the prevalence of vitamin D deficiency during the gestational period remains an alarmingly high global health problem. Taking into account the irreplaceable role of vitamin D in maternal and fetal health during pregnancy, it is crucial that all pregnant women maintain sufficient levels and preventive strategies are implemented to avoid the devastating effects of vitamin D deficiency [33,34].

During the last few decades, there has been a growing body of evidence highlighting the role of vitamin D in multiple brain functions and disorders. It has been observed that vitamin D receptors are expressed by neurons and glial cells throughout crucial brain regions associated with human behavior and cognition like thalamus, hippocampus, cerebral cortex, and amygdala. Animal studies have indicated the importance of vitamin D in fetal brain development by enhancing synaptogenesis and neuronal cell proliferation and migration while exerting neuroprotective and anti-inflammatory effects. On the other hand, vitamin D deficiency has been shown to promote neuronal inflammation and apoptosis, disruption of neurotransmitter signaling, alteration in the structural morphology of the brain and inevitably induce cognitive and behavioral alterations. Furthermore, vitamin D and its metabolites are involved in the regulation of various genes implicated in human brain development and proper function [35]. In adults, evidence supports the association of vitamin D deficiency with cognitive decline, and the development of neurodegenerative disorders like Parkinson’s and Alzheimer’s disease while vitamin D supplementations have been proven beneficial in counteracting the symptoms of these disorders in certain studies [36].

During childhood, recent evidence supports a strong association between low vitamin D levels and the development of autistic traits [37]. The effect of maternal vitamin D levels on subsequent neurodevelopmental outcomes in children has been greatly explored by various studies which have yielded contradictory results. Chi MZ et al. discovered a strong association between gestational maternal vitamin D levels with cognitive and motor development as well as anthropometric measurements at birth and at 6 months of age [23]. In their recent study, Rodgers et al. showed that children whose mothers received high doses of vitamin D supplementation and had sufficient levels of maternal vitamin D levels antenatally exhibited improved language skills at 3 to 5 years old neurodevelopmental assessment [30]. Similarly, Shekhawat et al. reported that at 6 months of age, infants of vitamin D-deficient mothers had lower scores in the cognitive domain while there was no other association found in the other neurodevelopmental domains [15]. The same study also elucidated a strong correlation between reduced cord blood vitamin D levels and social-emotional development at 9 months of age [15]. Another study by Cantio E et al., reported a notable effect of maternal hypovitaminosis D early in pregnancy on the intelligence quotient scores at the age of 7 years, especially in the male population, highlighting possible sex-related differences in the effect of antenatal vitamin D status [18].

Vitamin D supplementation during pregnancy and its benefits on maternal and neonatal health has been explored by various researchers, yet the results remain speculative. A recent meta-analysis of randomized controlled trials indicated a positive effect of antenatal vitamin D supplementation on offspring’s vitamin D levels and neonatal length as well as reduced rates of fetal and neonatal mortality. The same meta-analysis found no association between antenatal vitamin D supplementation and prematurity, birth weight, or head circumference [38]. In line with previous studies, our study found no association between maternal high-dose supplementation and improved neurodevelopmental outcomes. Sass L. et al. reported no significant differences between a high-dose regiment of vitamin D supplementation and a standard dose regiment given to pregnant women in the last semester of pregnancy with subsequent offspring neurodevelopment. Interestingly, high-dose vitamin D supplementation was correlated with deficits in language development of a 2-year-old child while the standard recommended dose showed no such correlation [9]. It is important to elucidate any potential interconnection between vitamin D supplementation during pregnancy and its benefits on maternal and neonatal health since any positive finding and correlation between these two, could potentially be extremely significant in the long-term when it comes to psychosocial health and health in general.

Future research should focus on whether vitamin D supplementation (schedule and dosage) could be considered before pregnancy and if longer follow-up periods may be necessary in order to detect significant changes. Furthermore, research should address specific populations (i.e., low-income), which requires clarification because one might argue that if there is a lack of positive association between vitamin D levels and fetal/maternal outcomes, then there is no need for vitamin D supplementation.

### Limitations

Concerning our findings and the evidence presented, we must acknowledge that our study faces inherent variability and our results need to be interpreted with caution. There is a high heterogeneity present in the majority of the included studies especially regarding the timing and the methods conducted for the neurodevelopmental assessment of children. To be more specific, the majority of studies used Bayley Scales Of Infant And Toddler Development, Third edition as their assessment tool. Bayley-III comprises five different subscales addressing cognition, language, motor, social-emotional, and adaptive behavior domains and evaluates children between 1 and 42 months of age. On the contrary, other studies used other screening tools which evaluate fewer neurodevelopmental domains like the Brigance Screen II or less sensitive methods for neurodevelopmental assessment like the parent-completed Ages and Stages Questionnaires, thus possibly influencing the results [27].

While in some studies the researchers performed neurodevelopmental assessment as early as at 6 months of age [15], in other studies neurodevelopmental outcomes were evaluated later in early childhood [30]. Additionally, the included studies addressed different aspects of neurodevelopmental evolution, therefore there was inevitable inconsistency between the domains of neurodevelopment in each study. In addition, early assessments may have failed to capture longer-term developmental effects, and later assessments may have introduced recall bias that may have altered the results.

Moreover, we also observed discrepancies in the timing and the methods for vitamin D measurement antenatally which may result in misclassification of our results. Of note, measurement assays differ between different vendors and so it is important to adequately label the methods of vitamin D measurements [39]. It is well-known that fetal brain development begins early in pregnancy and undergoes significant variations with different windows of susceptibility throughout the gestational period, therefore the timing of maternal vitamin D serum levels is crucial for the proper assessment of neurodevelopmental status. Furthermore, confounding variables and their adjustment differ among the studies we included in our study and specific confounders like the differing population under study may have impacted the result. Finally, differences in maternal baseline vitamin D status and definition of vitamin D deficiency across the different studies constitute another important limitation in our meta-analysis.

## 5. Conclusions

Overall, our study definitely supports the importance of vitamin D adequacy during pregnancy on various aspects of infant neurodevelopment, although the effect seems to be minimal and statistically non-significant. Nevertheless, the impact of prenatal vitamin D deficiency on offspring neurodevelopment constitutes a great challenge for researchers and definitely seeks further investigation. Future studies with longitudinal follow-up should incorporate the assessment of all domains of neurodevelopment at prespecified age cutoffs in order to minimize the heterogenicity and confounding factors present in the current literature and enable the extraction of concrete and valid conclusions. Additionally, the effect of vitamin D supplementation during pregnancy with specific doses and regimens should be explored in greater depth for its safety and efficacy. Measurement of vitamin D levels should be performed at multiple stages throughout the gestational period in order to identify the window that maternal vitamin D levels have the most profound effect on subsequent neurodevelopmental impairment.

There is a need for large cohorts and large-scale, multi-center randomized controlled trials, which would help guide future research that will evaluate spherically the cognitive outcomes of offspring in prespecified age cutoff, that will expand throughout optimal timespan and that will at least evaluate all neurodevelopmental outcomes (cognitive, motor, language, social, emotional, and behavioral), so as to avoid gaps in future knowledge. Finally, the conduction of larger cohort studies with a greater number of participants and multicenter randomized controlled trials is warranted, in order to provide specific recommendations for pregnant women and ensure optimal offspring neurodevelopment.

## Figures and Tables

**Table 1:** Methodological characteristics.

*Antenatal maternal Vitamin D levels*						
Year; Author	Study type	Patient number	Timing	Outcome	Outcome measures	Assessment scale
2024; Gao [14]	Prospective	746 mother–child pairs	second trimester	24 months	Cognitive, Language, Motor	BSID-III
2024; Shekhawat [15]	Prospective	175 mother–child pairs	Intrapartum period (36.02–40.4 weeks of gestation	6 and 9 months of age	cognitive, motor, language and social-emotional	BSID-III
2024; Thinggaard [16]	Prospective	944 mother–child pairs	GA < 20 weeks, GA ≥ 20 weeks and umbilical cord at delivery	5 years	ADHD symptoms (emotional, behavioral and social problems)	CBCL/1½-5
2023; Zhang [17]	Prospective	649 mother–infant pairs	in three trimesters, and cord blood at delivery	24 months	cognition, language, motor, social-emotional, and adaptive behaviour	BSID-III
2023; Cantio [18]	Prospective	1404 mother–child pairs	GA < 20 weeks or GA ≥ 20 weeks, from the umbilical cord	7 years	cognitive functions	WISC-V FSIQ
2021; Melough [19]	Prospective	1019 eligible dyads	16 and 28 weeks	4–6 Years	IQ (Neurocognitive Development)	SB5 VIQ NVIQ FSIQ
2020; Voltas [29]	Randomized	422 mother–infant pairs	12th and 36th weeks of gestation	0 to 42 months	-cognitive, language, and motor skills	BSID-III
2020; Dhamayanti [20]	Prospective	141 mother–infant pairs	10 and 14 weeks of gestational age	3, 6 and 12 months	gross motor, fine motor, communication, problem solving and personal–social domains	ASQ-3
2020; Windham [21]	Retrospective	534 ASD, 181 ID, 421 controls	mid-pregnancy	n/a	Autism or low IQ	-DSM-IV-TR -composite developmental/cognitive score < 70
2019; Sudfeld [22]	Prospective	257 HIV–infected mothers and their HIV–exposed uninfected infants	Third trimester	12 months +/− 3 months	neurodevelopment (cognitive, motor, and language)	BSID-III
2018; Chi [23]	Prospective	160 mother–child pairs	Over 28 weeks	6 months	-cognitive, motor, and language -socioemotional and adaptive behavior scores	-BSID-III -MDI -PDI
2017; Darling [24]	Prospective	7065 mother–child pairs	During pregnancy (Mean 23.4 weeks)	6 months–9 years	-behavioral development (81 months of age) -IQ (8 years of age)	-SDQ -Wechsler Intelligence Scale for Children
2017; Laird [25]	Prospective	202 healthy pregnant women	One day after delivery	5 years	birth weight and head circumference, neurocognitive function	FT, PLS-TL, PLS-VA, PLS-AC, WJSAT, CBCL, KBIT-VK, KBIT-M
2012; Whitehouse [26]	Prospective	743 women	18 weeks	2-, 5-, 8-, 10-, 14-, 17-years	neurodevelopment behavioral, emotional, and language	CBCL
2008; Gale [4]	Prospective	596 women	Third trimester	9 months, 9 years	-Cognitive function -Psychological health	-WASI -SDQ
*Vitamin D supplementation*						
2023; Rodgers [30]	Randomized	350 women	From 12–16 weeks of gestation until delivery	3–5 years	language, motor, and academic	Brigance Screen II
2023; Vestergaard [31]	Randomized	70 pregnancies	late first trimester to delivery	time of delivery	genes involved in pathways linked to autism spectrum disorders, ADHD, schizophrenia	next-generation RNA sequencing
2020; Sass [9]	Randomized	623 women	From 24 weeks until 1 week postpartum	6 years	-Cognitive at 2.5 y.o. -motor milestone achievement -language at age 3 y.o. -emotional/behavioural at 6 y.o.	BSID-III, Denver Developmental Index, WHO milestone registration, MacArthur-Bates Communicative Development Inventories, ASQ, SDQ
*Fetal Vitamin D and neurodevelopment*						
2018; Wang [27]	Prospective	1244 infants	At delivery	6 months, 1 and 2 years	communication, gross motor, fine motor, problem-solving skills, and personal social)	ASQ
2017; Gould [32]	Randomized	337 infants	At delivery	−18 months −4 years	cognitive, language and motor	-BSID-III -DAS II and CELF-P2

## References

[1] Wimalawansa S.J. (2024). Physiology of Vitamin D—Focusing on Disease Prevention. Nutrients.

[2] Chien M.-C., Huang C.-Y., Wang J.-H., Shih C.-L., Wu P. (2024). Effects of vitamin D in pregnancy on maternal and offspring health-related outcomes: An umbrella review of systematic review and meta-analyses. Nutr. Diabetes.

[3] Berridge M.J. (2018). Vitamin D deficiency: Infertility and neurodevelopmental diseases (attention deficit hyperactivity disorder, autism, and schizophrenia). Am. J. Physiol.-Cell Physiol..

[4] Gale C.R., Robinson S.M., Harvey N.C., Javaid M.K., Jiang B., Martyn C.N., Godfrey K.M., Cooper C., The Princess Anne Hospital Study Group (2008). Maternal vitamin D status during pregnancy and child outcomes. Eur. J. Clin. Nutr..

[5] Palacios C., Gonzalez L. (2014). Is vitamin D deficiency a major global public health problem?. J. Steroid Biochem. Mol. Biol..

[6] Eyles D., Brown J., Mackay-Sim A., McGrath J., Feron F. (2003). Vitamin d3 and brain development. Neuroscience.

[7] Becker A., Eyles D.W., McGrath J.J., Grecksch G. (2005). Transient prenatal vitamin D deficiency is associated with subtle alterations in learning and memory functions in adult rats. Behav. Brain Res..

[8] Tuovinen S., Räikkönen K., Holmlund-Suila E., Hauta-alus H., Helve O., Rosendahl J., Enlund-Cerullo M., Kajantie E., Valkama S., Viljakainen H. (2021). Effect of High-Dose vs Standard-Dose Vitamin D Supplementation on Neurodevelopment of Healthy Term Infants: A Randomized Clinical Trial. JAMA Netw. Open.

[9] Sass L., Vinding R.K., Stokholm J., Bjarnadóttir E., Noergaard S., Thorsen J., Sunde R.B., McGrath J., Bønnelykke K., Chawes B. (2020). High-Dose Vitamin D Supplementation in Pregnancy and Neurodevelopment in Childhood: A Prespecified Secondary Analysis of a Randomized Clinical Trial. JAMA Netw. Open.

[10] García-Serna A.M., Morales E. (2020). Neurodevelopmental effects of prenatal vitamin D in humans: Systematic review and meta-analysis. Mol. Psychiatry.

[11] Daraki V., Roumeliotaki T., Koutra K., Chalkiadaki G., Katrinaki M., Kyriklaki A., Kampouri M., Margetaki K., Vafeiadi M., Papavasiliou S. (2018). High maternal vitamin D levels in early pregnancy may protect against behavioral difficulties at preschool age: The Rhea mother–child cohort, Crete, Greece. Eur. Child Adolesc. Psychiatry.

[12] Lancaster M.A. (2024). Unraveling mechanisms of human brain evolution. Cell.

[13] Sterne J.A., Hernán M.A., Reeves B.C., Savović J., Berkman N.D., Viswanathan M., Henry D., Altman D.G., Ansari M.T., Boutron I. (2016). ROBINS-I: A tool for assessing risk of bias in non-randomised studies of interventions. BMJ.

[14] Gao Y., Zhang Y., Luo J., Mao D., Lei X., Liu C., Zhang S., Yao Q., Li J., Zhang J. (2024). Effect modification by maternal vitamin D status in the association between prenatal exposure to per- and polyfluoroalkyl substances and neurodevelopment in 2-year-old children. Environ. Int..

[15] Shekhawat D.S., Singh K., Singh P., Vyas V., Varthya S.B., Sharma P. (2024). Prenatal vitamin D levels and infant cognitive, motor, language and social-emotional development at 6 and 9 months of age. Nutr. Neurosci..

[16] Thinggaard C.M., Dalgård C., Möller S., Christesen H.B.T., Bilenberg N. (2024). Vitamin D status in pregnancy and cord blood is associated with symptoms of attention-deficit hyperactivity disorder at age 5 years: Results from Odense Child Cohort. Aust. N. Z. J. Psychiatry.

[17] Zhang Y., Zhou C.-Y., Wang X.-R., Jiao X.-T., Zhang J., Tian Y., Li L.-L., Chen C., Yu X.-D. (2023). Maternal and neonatal blood vitamin D status and neurodevelopment at 24 months of age: A prospective birth cohort study. World J. Pediatr..

[18] Cantio E., Bilenberg N., Nørgaard S.M., Beck I.H., Möller S., Cantio C., Jensen T.K., Mortensen N.B., Rasmussen A., Christesen H.B.T. (2023). Vitamin D status in pregnancy and childhood associates with intelligence quotient at age 7 years: An Odense child cohort study. Aust. N. Z. J. Psychiatry.

[19] Melough M.M., Murphy L.E., Graff J.C., Derefinko K.J., LeWinn K.Z., Bush N.R., Enquobahrie D.A., Loftus C.T., Kocak M., Sathyanarayana S. (2021). Maternal Plasma 25-Hydroxyvitamin D during Gestation Is Positively Associated with Neurocognitive Development in Offspring at Age 4–6 Years. J. Nutr..

[20] Dhamayanti M., Noviandhari A., Supriadi S., Judistiani R.T., Setiabudiawan B. (2020). Association of maternal vitamin D deficiency and infants’ neurodevelopmental status: A cohort study on vitamin D and its impact during pregnancy and childhood in Indonesia. J. Paediatr. Child Health.

[21] Windham G.C., Pearl M., Poon V., Berger K., Soriano J.W., Eyles D., Lyall K., Kharrazi M., Croen L.A. (2020). Maternal Vitamin D Levels During Pregnancy in Association With Autism Spectrum Disorders (ASD) or Intellectual Disability (ID) in Offspring; Exploring Non-linear Patterns and Demographic Sub-groups. Autism Res..

[22] Sudfeld C.R., Jacobson D.L., Rueda N.M., Neri D., Mendez A.J., Butler L., Siminski S., Hendricks K.M., Mellins C.A., Duggan C.P. (2019). Third Trimester Vitamin D Status Is Associated With Birth Outcomes and Linear Growth of HIV-Exposed Uninfected Infants in the United States. JAIDS J. Acquir. Immune Defic. Syndr..

[23] Chi M.-Z., Zhu L., Zhang Z.-L., Jin F.-F., Shao H.-R., Zheng J.-Y., Wu C., Hu G.-Q. (2018). The Relationship between Maternal Serum Vitamin D Levels and Infant Neurodevelopment and Anthropometry: A Prospective Observational Study. J. Nutr. Sci. Vitaminol..

[24] Darling A.L., Rayman M.P., Steer C.D., Golding J., Lanham-New S.A., Bath S.C. (2017). Association between maternal vitamin D status in pregnancy and neurodevelopmental outcomes in childhood: Results from the Avon Longitudinal Study of Parents and Children (ALSPAC). Br. J. Nutr..

[25] Laird E., Thurston S., Van Wijngaarden E., Shamlaye C., Myers G., Davidson P., Watson G., McSorley E., Mulhern M., Yeates A. (2017). Maternal Vitamin D Status and the Relationship with Neonatal Anthropometric and Childhood Neurodevelopmental Outcomes: Results from the Seychelles Child Development Nutrition Study. Nutrients.

[26] Whitehouse A.J.O., Holt B.J., Serralha M., Holt P.G., Kusel M.M.H., Hart P.H. (2012). Maternal Serum Vitamin D Levels During Pregnancy and Offspring Neurocognitive Development. Pediatrics.

[27] Wang H., Yu X.D., Huang L.S., Chen Q., Ouyang F.X., Wang X., Zhang J. (2018). Fetal vitamin D concentration and growth, adiposity and neurodevelopment during infancy. Eur. J. Clin. Nutr..

[28] Sterne J.A.C., Savović J., Page M.J., Elbers R.G., Blencowe N.S., Boutron I., Cates C.J., Cheng H.-Y., Corbett M.S., Eldridge S.M. (2019). RoB 2: A revised tool for assessing risk of bias in randomised trials. BMJ.

[29] Voltas N., Canals J., Hernández-Martínez C., Serrat N., Basora J., Arija V. (2020). Effect of Vitamin D Status during Pregnancy on Infant Neurodevelopment: The ECLIPSES Study. Nutrients.

[30] Rodgers M.D., Mead M.J., McWhorter C.A., Ebeling M.D., Shary J.R., Newton D.A., Baatz J.E., Gregoski M.J., Hollis B.W., Wagner C.L. (2023). Vitamin D and Child Neurodevelopment—A Post Hoc Analysis. Nutrients.

[31] Vestergaard A.L., Andersen M.K., Olesen R.V., Bor P., Larsen A. (2023). High-Dose Vitamin D Supplementation Significantly Affects the Placental Transcriptome. Nutrients.

[32] Gould J.F., Anderson A.J., Yelland L.N., Smithers L.G., Skeaff C.M., Zhou S.J., Gibson R.A., Makrides M. (2017). Association of cord blood vitamin D with early childhood growth and neurodevelopment. J. Paediatr. Child Health.

[33] Zhang H., Wang S., Tuo L., Zhai Q., Cui J., Chen D., Xu D. (2022). Relationship between Maternal Vitamin D Levels and Adverse Outcomes. Nutrients.

[34] Mansur J.L., Oliveri B., Giacoia E., Fusaro D., Costanzo P.R. (2022). Vitamin D: Before, during and after Pregnancy: Effect on Neonates and Children. Nutrients.

[35] Gáll Z., Székely O. (2021). Role of Vitamin D in Cognitive Dysfunction: New Molecular Concepts and Discrepancies between Animal and Human Findings. Nutrients.

[36] Sailike B., Onzhanova Z., Akbay B., Tokay T., Molnár F. (2024). Vitamin D in Central Nervous System: Implications for Neurological Disorders. Int. J. Mol. Sci..

[37] Wang Z., Ding R., Wang J. (2020). The Association between Vitamin D Status and Autism Spectrum Disorder (ASD): A Systematic Review and Meta-Analysis. Nutrients.

[38] Liu Y., Ding C., Xu R., Wang K., Zhang D., Pang W., Tu W., Chen Y. (2022). Effects of vitamin D supplementation during pregnancy on offspring health at birth: A meta-analysis of randomized controlled trails. Clin. Nutr..

[39] Snellman G., Melhus H., Gedeborg R., Byberg L., Berglund L., Wernroth L., Michaëlsson K. (2010). Determining Vitamin D Status: A Comparison between Commercially Available Assays. PLoS ONE.

